# Clinical features and prognosis of double primary malignant neoplasms in patients with non-hodgkin lymphoma

**DOI:** 10.1007/s12672-023-00667-6

**Published:** 2023-05-03

**Authors:** Zhumei Zhan, Wei Guo, Jia Li, Xin Wan, Jing Guo, Ou Bai

**Affiliations:** Department of Hematology, The First Hospital of Jilin University, No. 71 Xinmin Street, Chaoyang District, Changchun, Jilin 130021 China

**Keywords:** Double primary malignant tumors, Non-Hodgkin lymphoma, Clinical features, Prognosis

## Abstract

To investigate the clinical features, survival, and prognostic factors of patients with double primary malignant neoplasms (DPMNs) comprising non-Hodgkin lymphoma (NHL) and malignant solid tumors. Of the 2352 patients diagnosed with NHL, 105 (4.46%) patients were diagnosed with DPMNs, 42 (40.0%) had NHL first (the NHL-first group) and 63 (60.0%) had solid tumor first (the ST-first group). Females were more frequent in the ST-first group, and the interval time between the two tumors was longer. More NHLs in early stages and originating from extranodal sites were observed in the NHL-first group. Male, age ≥ 55 years at diagnosis of the first tumor, interval time <60 months, NHL diagnosed first, NHL arising from an extranodal site, DPMNs without breast cancer, and no surgery for the first primary tumor were associated with poorer overall survival (OS). Interval time <60 months and NHL diagnosed first were independent risk factors that affected the prognosis of patients with DPMNs. Therefore, careful monitoring and follow-up are especially important for these patients. 50.5% (53/105) of patients with DPMNs did not receive chemotherapy or radiotherapy prior to the diagnosis of the second tumor. We further compared the baseline characteristics of diffuse large B-cell lymphoma(DLBCL) patients with and without solid tumors, the former had a higher proportion of extranodal DLBCL, suggesting that extranodal DLBCL is more likely to develop solid tumors than nodal DLBCL.

## Introduction

The concept of multiple primary malignant neoplasms (MPMNs) was first introduced by Warren and Gates in 1932 and refers to the occurrence of two or more independent primary malignant neoplasms in the single or multiple organ tissues of the same patient simultaneously or sequentially [1]. The incidence of MPMNs has been reported to range from 0.73 to 11.7% in various studies from different countries [2–4]. Among MPMNs, double primary malignant neoplasms (DPMNs) are more common, whereas three or more tumors are less common.

Non-Hodgkin lymphoma (NHL) combined with malignant solid tumors represent a unique subtype of DPMNs. With the advancements in diagnostic procedures and the continuous development of new drugs, the overall survival (OS) of patients with NHL has been prolonged [5], allowing more patients to survive long enough to be at risk of development of a second cancer. Some patients with other malignancies may also develop NHL during the course of their disease. Both conditions further aggravate the disease process and significantly affect the patient’s prognosis [6–8]. Most studies on DPMNs have been case reports and epidemiological investigations. Whether NHL is intrinsically associated with other malignancies or with certain treatment options is of great interest, but few studies have been reported in this area. In the present study, we retrospectively summarized the clinical features, treatment, and prognostic factors affecting patients with DPMNs comprising NHL and malignant solid tumors, aiming to improve our understanding and provide a basis for further exploration of the underlying pathogenetic mechanisms.

## Patients and methods

### Data collection

The clinical data of all patients diagnosed with NHL at the First Hospital of Jilin University between July 2011 to July 2021 were reviewed. Patients were included if they met the diagnostic criteria for MPMNs proposed by Warren and Gates [1]:(1) each tumor is malignant, (2) each tumor has its own pathological features, and (3) the diagnosis of metastatic or recurrent tumors can be excluded. NHL was diagnosed and classified according to the 2008 WHO classification of lymphoid tumors, and malignant solid tumors were classified according to the 7th edition of the American Joint Committee on Cancer (AJCC). All patients were ≥ 14 years of age at the onset and both primary tumors were pathologically confirmed. Ultimately, 105 patients with DPMNs consisting of NHL and malignant solid tumors were included. On the basis of which tumor occurred first, we divided the patients into the NHL-first group and solid-tumor-first (ST-first) group. The study was approved by the Clinical Research Management Committee (2022-KS-069) and the Human Ethics Committee of the First Hospital of Jilin University (2022 − 545).

### Treatment and follow-up

Patients with indolent stage I-II NHL are treated with local therapy (surgery, local radiotherapy)or a wait-and-watch approach. Patients with aggressive NHL and indolent stage III–IV NHL are treated with chemotherapy-based regimens, with or without combined radiotherapy and targeted therapy according to guidelines [9]. For malignant solid tumors, treatment options include surgical resection, radiotherapy, and chemotherapy, depending on the tumor classification and stage as well as the individual patient’s circumstances.

The patients’ follow up conditions were carried out by telephone, outpatient department visits, or electronic medical records. OS was defined as the period from the date of diagnosis of the first primary tumor to death or the end of follow-up (September 2021).

### Statistical analysis

SPSS (version 26.0; SPSS, Chicago, IL, USA) was used for all statistical analyses. Group differences were evaluated using the chi-square or Fisher exact test. The Kaplan–Meier method was used to plot survival curves, and the log-rank test was used to compare differences between groups. Cox proportional hazard multivariate analysis was performed to identify independent factors associated with prognosis. All P values were two-sided, and P = 0.05 or less was considered statistically significant.

## Results

### Clinical features of patients with DPMNs

**Fig. 1:**
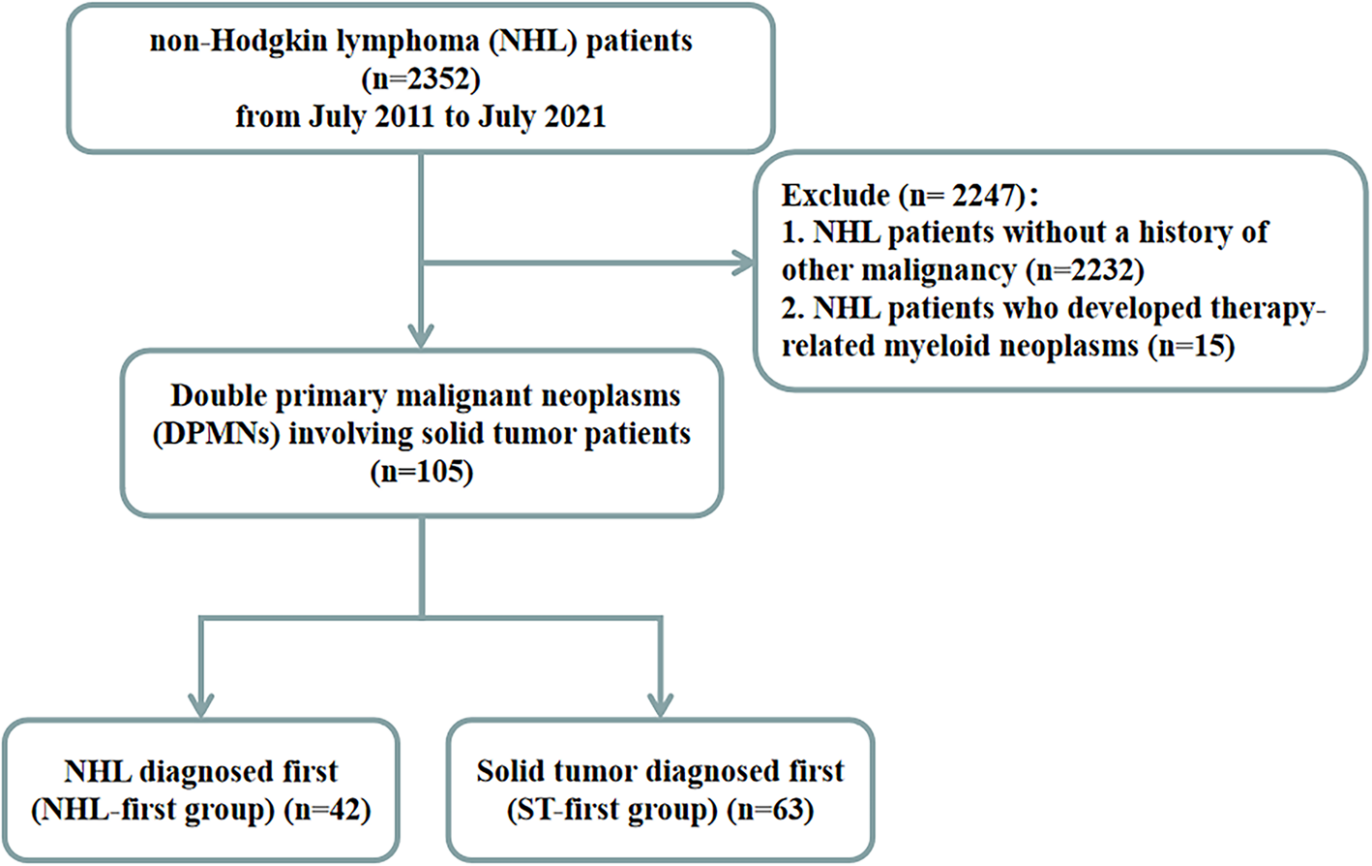
Flow chart of the present study

Of the 2352 patients diagnosed with NHL in our center during the study period, 105 (4.46%) patients (52 men and 53 women) were diagnosed with DPMNs comprising NHL and malignant solid tumors. Of them, 42 (40.0%) patients were in the NHL-first group, and 63 (60.0%) patients were in the ST-first group (Fig. 1). The median age of the patients was 60 (50–66) years at the diagnosis of the first tumor and 62 (56–70) years at the diagnosis of the second tumor. The median interval time between the two tumors was 26 months.

**Fig. 2:**
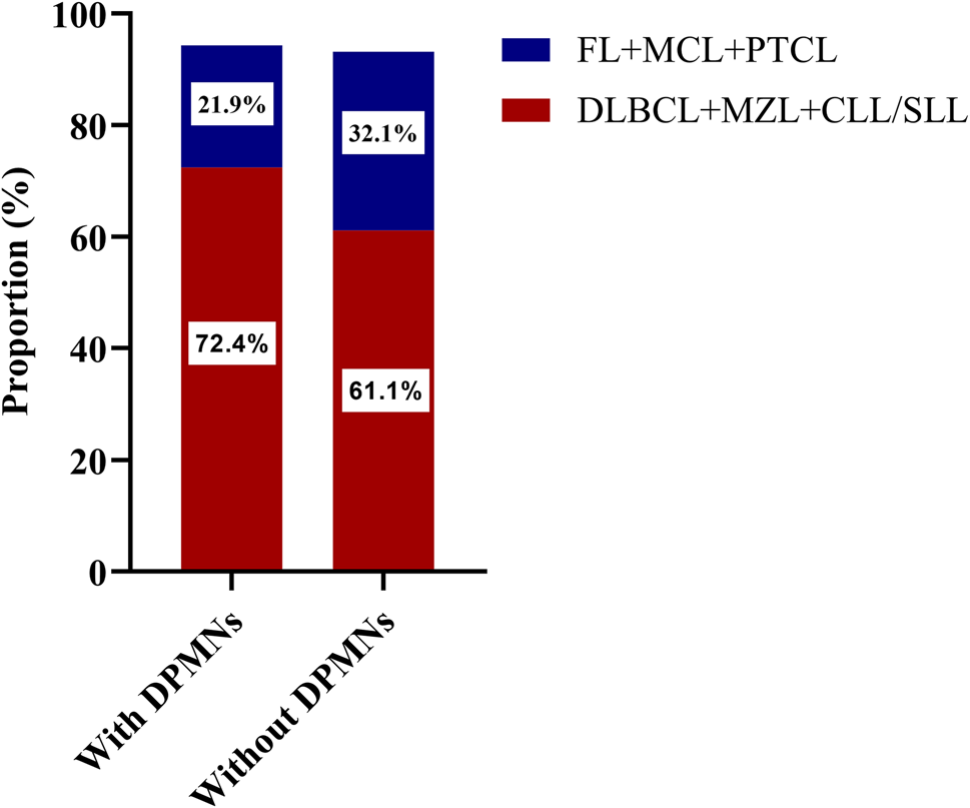
Distribution of NHL subtypes with DPMNs and without DPMNs during 2011–2021

Diffuse large B-cell lymphoma(DLBCL) (51.4%) was the most frequent pathological type of NHL among patients with DPMNs. Moreover, compared with NHL patients without DPMNs, those with DPMNs had a higher total proportion of DLBCL, marginal zone lymphoma (MZL), and chronic lymphocytic leukemia/small lymphocytic lymphoma (CLL/SLL) (72.4% vs. 61.1%) and a lower total proportion of follicular lymphoma (FL), mantle cell lymphoma (MCL), and peripheral T-cell lymphoma (PTCL) (21.9% vs. 32.1%) (P = 0.002; Fig. 2).

### Comparison of clinical features of the NHL-first and ST-first groups

The clinical features of the NHL-first and ST-first groups are presented in Tables 1 and 2. Females were more frequent in the ST-first group (P = 0.004). 37 (88.1%) patients have a short interval time (<60 months) in the NHL-first group. Compared with the ST-first group, lymphoma was diagnosed at earlier stages in the NHL-first group; 26 (61.9%) patients were diagnosed at stage I/II, and 33(78.6%)patients with IPI score ≤ 2. Additionally, the proportion of patients with solid tumors diagnosed as stage I/II was lower (71.5% vs. 87.3%).

Regarding the distribution and pathology of DPMNs, there were more patients whose lymphoma originating from extranodal sites in the NHL-first group, and the gastrointestinal tract was the most common site. DLBCL (45.2%), CLL/SLL (14.63%) and PTCL (14.63%) were more frequently seen in the NHL-first group; DLBCL (55.6%) and MZL (11.1%) were more common in the ST-first group. In the NHL-first group, the most frequent sites of solid tumors were the digestive system (40.5%) and respiratory system (35.7%), whereas in the ST-first group, the most common sites were breast (28.6%), the digestive system (27.0%), and thyroid (19.0%). Adenocarcinomas were the most common pathological type of solid tumor in both groups.

**Table 1:** Comparison of general characteristics and NHL features between the NHL-first and ST-first groups

Variable	NHL-first group n (%)	ST-first group n (%)	*P* value
Gender			0.004
Male	28(66.7)	24(38.1)	
Female	14(33.3)	39(61.9)	
Age at diagnosis of the primary tumor, y	61(57–66)	56(46–64)	0.044
Age at diagnosis of the second tumor, y	62(58–67)	63(55–70)	0.796
Interval between two tumors, m			<0.001
<60	37(88.1)	31(49.2)	
≥60	5(11.9)	32(50.8)	
β2-MG			0.750
Normal	20(47.6)	32(50.8)	
Elevated	22(52.4)	31(49.2)	
Hemoglobin			0.518
<110 g/L	9(21.43)	17(27.0)	
≥ 110 g/L	33(78.57)	46(73.0)	
Ann Arbor stage			0.017
I/II	26(61.9)	24(38.1)	
III/IV	16(38.1)	39(61.9)	
B symptoms			0.612
Yes	9(21.4)	11(18.37)	
No	33(78.6)	52(81.63)	
IPI score			0.035
0–2	9(21.4)	26(41.3)	
3–5	33(78.6)	37(58.7)	
Primary sites of NHL			0.008
Nodal	13(31.0)	36(57.1)	
Extranodal	29(69.0)	27(42.9)	
Pathological type of NHL			0.716*
DLBCL	20(47.6)	35(55.6)	
MZL	4(9.5)	7(11.1)	
CLL/SLL	6(14.3)	5(7.9)	
FL	4(9.5)	5(7.9)	
MCL	0	2(3.2)	
PTCL	6(14.3)	6(9.5)	
Others	2(4.8)	3(4.8)	

*Fisher test

IPI: International Prognostic Index

**Table 2:** Comparison of solid tumor features between the NHL-first and ST-first groups

Variable	NHL-first group n (%)	ST-first group n (%)	*P* value
Sites of solid tumor			0.007*
Breast	3(7.1)	18(28.6)	
Thyroid	4(9.5)	12(19.0)	
Digestive system	17(40.5)	17(27.0)	
Esophagus	1(2.4)	0	
Stomach	5(11.9)	6(9.5)	
Colon	4(9.5)	2(3.2)	
Rectum	1(2.4)	5(7.9)	
Liver	6(14.3)	3(4.8)	
Pancreas	0	1(1.6)	
Respiratory system	15(35.7)	9(14.3)	
Larynx	1(2.4)	2(3.2)	
Lung	14(33.3)	7(11.1)	
Urinary system	0	3(4.8)	
Others	3(7.1)	4(6.3)	
Pathological type of solid tumor			0.082*
Adenocarcinoma	27(64.3)	50(79.4)	
Squamous carcinoma	5(11.9)	5(7.9)	
Hepatocellular carcinoma	6(14.3)	3(4.8)	
Neuroendocrine carcinoma	1(2.4)	0	
Small cell carcinoma	2(4.8)	0	
Clear cell carcinoma	0	3(4.8)	
Others	1(2.4)	2(3.2)	
Clinical stage of solid tumor			0.160*
I	17(40.5)	25(39.7)	
II	13(31.0)	30(47.6)	
III	8(19.0)	6(9.5)	
IV	4(9.5)	2(3.2)	

*Fisher test

### Treatment of DPMNs

Among the 105 patients with DPMNs, 95 (90.5%) patients accepted therapy after the diagnosis of the first tumor, and 92 (87.6%) were treated after the diagnosis of the second tumor. In terms of NHL treatment, 31 (73.8%) patients in the NHL-first group and 51 (81.0%) patients in the ST-first group received chemotherapy alone or chemotherapy-based combination therapy. For malignant solid tumor treatment, 25 (59.5%) patients in the NHL-first group and 55 (87.3%) patients in the ST-first group accepted surgery alone or surgery-based treatment (Table 3). In addition, 50.5% (53/105) of patients did not receive chemotherapy or radiotherapy prior to the diagnosis of the second tumor.

**Table 3:** Treatment conditions of 105 DPMNs patients

Treatment	NHL-first group (n = 42)		ST-first group (n = 63)	
	NHL	Solid tumor	Solid tumor	NHL
Surgery	3(7.2)	21(50.0)	33(52.4)	5(7.9)
Chemotherapy	21(50.0)	7(16.7)	4(6.3)	48(76.2)
Chemotherapy + radiotherapy	4(9.5)	2(4.8)	0	1(1.6)
Surgery + chemotherapy	6(14.3)	4(9.5)	18(28.6)	1(1.6)
Surgery + radiotherapy	0	0	4(6.3)	1(1.6)
Endocrine therapy	0	2(4.8)	2(3.2)	0
Without treatment	8(19.0)	6(14.3)	2(3.2)	7(11.1)

### Survival outcomes

**Fig. 3:**
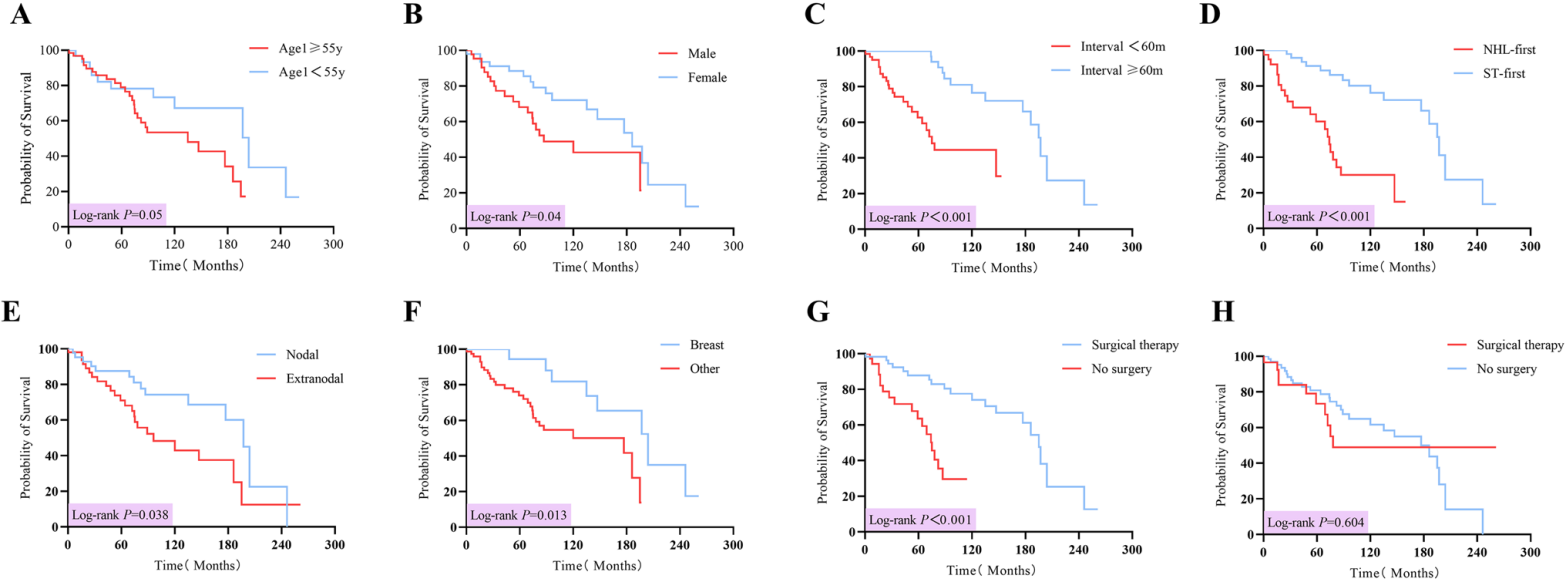
**A**–**H** Comparison of survival time with different subgroups for DPMNs patients. **A** The survival times of younger people (<55 years old) and older people (≥ 55 years old) at the diagnosis of the first primary cancer. **B** The survival times for male and female. **C** The survival times for different interval times (<60 vs. ≥ 60 months). **D** The survival times of DPMNs patients with NHL diagnosed first and solid tumor diagnosed first. **E** The survival times of DPMNs patients with NHL arose from nodal site and extranodal site. **F** The survival times for DPMNs patients involving breast and other systems tumor. **G** The survival times for surgical therapy and no surgery for the first primary cancer. **H** The survival times for surgical therapy and no surgery for the second primary cancer

Up to September 2021, 96 patients with DPMNs were successfully followed up with a median period of 93 months from the diagnosis of the first primary tumor, and 37 patients died, including 21 in the NHL-first group and 16 in the ST-first group. The median survival time of these 96 patients was 177 months (95% CI = 114.5–239.4) from the diagnosis of the first primary tumor. Kaplan–Meier survival curves showed that the cumulative survival rates of patients with DPMNs were 78.8% and 57.8% at 5 and 10 years, respectively. The OS rates at 5 and 10 years were 60.1% and 30.1% in the NHL-first group, and 91.2% and 76.1% in the ST-first group, respectively. Patients in the ST-first group had better OS than those in the NHL-first group (P <0.001) (Fig. 3D).

For all patients with DPMNs, factors such as male, age ≥ 55 years at diagnosis of the first tumor, interval time <60 months, NHL diagnosed first and no surgery for the first primary tumor were associated with poorer OS. There was a statistically significant and better OS in DPMNs patients with NHL arising from a nodal site or involving breast cancer (Fig. 3). Multivariate Cox proportional hazard regression analysis showed that interval time <60 months and NHL diagnosed first were independent risk factors that affected the prognosis of patients with DPMNs (Table 4).

**Table 4:** Analysis of risk factors influencing overall survival of DPMNs patients

Varaible	Univariate analysis		Multivariate analysis	
	*P* value	HR (95%CI)	*P* value	HR (95%CI)
Male vs. Female	0.044	2.032 (1.019–4.049)	0.560	1.275 (0.563–2.887)
Age1 ≥ 55y	0.056	2.133 (0.980–4.640)		
Interval time<60 months	<0.001	4.688 (2.023–10.867)	0.041	2.578 (1.037–6.408)
Elevated β2-MG	0.044	2.065 (1.018–4.189)		
Hemoglobin <110 g/L	0.080	0.546 (0.277–1.076)		
Stage III/IV vs. I/II	0.436	1.302 (0.670–2.531)		
NHL diagnosed first	<0.001	5.882 (2.697–12.826)	0.037	2.830 (1.067–7.502)
Extranodal vs.Nodal	0.043	2.008 (1.023–3.941)	0.550	1.254 (0.597–2.636)
Involving breast cancer	0.019	0.310 (0.117–0.824)	0.323	0.546 (0.165–1.812)
Treatment for the first primary cancer (surgical therapy vs.no surgery)	<0.001	0.208 (0.093–0.462)	0.252	0.563 (0.210–1.506)

Age1: Age at diagnosis of the first malignancy

### Risk of malignant solid tumors in DLBCL survivors

We observed that patients have a poor prognosis in the NHL-first group, with DLBCL being the most common subtype. Therefore, we further compared the baseline characteristics of DLBCL patients with and without secondary solid tumors, the former had a higher proportion of extranodal DLBCL (85.0% vs. 24.0%) (P<0.001; Table 5).

**Table 5:** Comparison of baseline characteristics of DLBCL patients with and without secondary solid tumor

Characteristics	With secondary solid tumor (n = 20)	Without secondary solid tumor (n = 939)	*P* value
Gender			0.272
Male	13(65.0)	494(52.6)	
Female	7(35.0)	445(47.4)	
Age at diagnosis of DLBCL, y			0.121
<60	6(30.0)	493(52.5)	
≥60	14(70.0)	446(47.5)	
Subtype			0.226
GCB	3(15.0)	206(21.9)	
Non-GCB	15(75.0)	553(58.9)	
β2-MG			0.443
Normal	9(45.0)	319(34.0)	
High	11(55.0)	552(58.8)	
Ann Arbor stage			0.419
I/II	11(55.0)	431(45.9)	
III/IV	9(45.0)	508(54.1)	
B symptoms			0.821
Yes	6(30.0)	277(29.5)	
No	14(70.0)	662(70.5)	
IPI score			0.593
0–2	6(30.0)	336(35.8)	
3–5	14(70.0)	603(64.2)	
Primary sites of DLBCL			<0.001
Nodal	3(15.0)	714(76.0)	
Extranodal	17(85.0)	225(24.0)	
Chemotherapy or Radiotherapy			0.407
Yes	17(85.0)	850(90.5)	
NO	3(15.0)	89(9.5)	

*IPI* International Prognostic Index

## Discussion

DPMNs comprising NHL and solid tumors represent a unique subtype; it is easily confused with metastasis or recurrence of the first tumor in clinical practice. It often requires careful pathological biopsies and comprehensive consideration by the clinician to confirm the diagnosis. At our institute, 105 patients were diagnosed with DPMNs comprising NHL and malignant solid tumors over 10 years, with an incidence rate of 4.46% among patients with NHL, higher than the incidence of MPMTs in previous Chinese clinical studies [4, 10]. The median age at diagnosis of both tumors was < 65 years, and 37 (35.2%) patients have a longer interval time (≥ 60 months), indicating that young people are more likely to have second tumors and should be closely monitored for longer periods.

NHL, whether as the first or second primary tumor, is predominantly DLBCL. DLBCL is the most common subtype of NHL, representing approximately 30–40% of all cases in different geographic regions [11]. We observed differences in the distribution of solid tumors between the NHL-first group and ST-first group. The digestive system and respiratory system were the most common sites for solid tumors as the second tumor. In China, de novo NHL occurs more frequently in men than in women. In addition, the respiratory system and digestive system were the top 2 sites affected by solid tumors in men in China. This finding implies that during the follow-up of patients with NHL, these systems should be monitored for the development of solid tumors. The most frequent type of solid tumor as the first primary tumor was breast cancer. Breast cancer is a highly prevalent solid tumor with a longer survival time due to its low aggressiveness of the disease and effective combination treatments (e.g., surgery, radiotherapy, and endocrine therapy). A large sample study has shown that the development of breast cancer was associated with an increased risk of NHL other than anaplastic large cell lymphoma (ALCL), particularly in patients receiving hormone therapy and in younger patients [12]. It was previously thought that estrogen had a protective function against lymphomagenesis, conversely, anti-hormonal therapy such as tamoxifen has been shown to increase the risk of NHL [13].

We observed that more NHLs in the NHL-first group arose from extranodal sites, with the gastrointestinal tract being the most common. This predisposition suggests that NHLs arising from extranodal sites are more likely to develop a second tumor. This conclusion was also supported by our comparison of baseline characteristics between DLBCL patients with and without secondary solid tumors. A study based on the SEER database indicated that patients with extranodal DLBCL had an 18% higher risk of developing a second tumor compared to the general population, and that the initial site of DLBCL may predict the type of second tumor [14]. Another study also reported that extranodal involvement was significantly associated with an increased risk of second malignancy [6]. Furthermore, NHLs originating from the gastrointestinal tract may be susceptible to *Helicobacter pylori* infection, which has been linked to the development of gastric lymphoma as well as gastric cancer by inducing excessive activation and proliferation of lymphocytes [15, 16].

The pathogenesis of DPMNs remains unclear. Radiotherapy and chemotherapy were previously considered to be important risk factors for the development of second primary tumors. For instance, NHL patients treated with CHOP (cyclophosphamide, adriamycin, vincristine, and prednisone) chemotherapy regimens exhibited a significantly increased risk of lung and colorectal cancers [17]. Indolent NHL and MCL patients treated with bendamustine-rituximab (BR) had higher incidences of skin cancer and late infections compared to those treated with R-CHOP/R-CVP [18]. Therapy-related solid tumors typically occur at intervals longer than 7 years and are associated with the use of radiotherapy [19]. Xu et al. observed that patients with NHL undergoing combination therapy who received a field radiation dose ≥ 40 Gy had a significantly increased risk of lung, breast, and bladder cancer [20].

However, about half of the patients in our study had not received the above therapy prior to diagnosis of the second tumor, and the median interval between the two tumors was only 26 months. Therefore, we should pay greater attention to the intrinsic biology and host factors of NHL and solid tumors. Genetic predisposition and shared etiologic exposure might play a vital role in carcinogenesis. Singhal et al. analyzed 45 known cancer predisposition genes in germline samples of 202 patients with hematological malignancies plus one or more other primary cancers. The frequency of pathogenic germline variants in patients with lymphoid hematological malignancies was 19%, involving *CHEK2, BRCA1, DDX41*, and *TP53* [21]. Germline alternations in *BRCA2, ATM, BRIP1*, and *PALB2* have also been reported to affect the development and progression of MPMNs [22]. The three MZL subtypes share recurrent trisomies of chromosome 3 [23]; because several suppressor genes are located on the short arm of chromosome 3, this chromosomal instability may affect more solid cancers. Shared etiologic factors, including infectious agents, smoking, and obesity, may not only contribute to the development of malignant solid tumors but also affect the specific NHLs. For instance, *Helicobacter pylori*, hepatitis C virus, and HIV/AIDS, which are establid causes of some solid carcinomas, including stomach, liver, and anus cancers as well as Kaposi sarcoma, are also associated with the development of DLBCL, MZL, and Burkitt lymphoma [15]. This pattern of association should be further explored through the bidirectional analysis of NHL subtypes and malignant solid tumors. Intrinsic biological properties of NHL such as immune dysfunction might also drive the development of a second tumor. A multifactorial analysis revealed that lower baseline CD8 counts were associated with an increased risk of a second tumor in CLL/SLL patients [24], reinforcing that impaired immune surveillance is an important risk factor for MPMNs.

Our findings suggest that a shorter OS was observed in the NHL-first group than in the ST-first group. Several possible causes could account for this. First, in our cohort, NHL was predominantly secondary to solid tumors of the digestive and respiratory systems, which are highly aggressive, and the OS of these patients, even in the absence of NHL, is relatively short [25]. Second, the interval time between the two tumors in the NHL-first group is shorter, reflecting the overlapping adverse effect of both malignancies on survival. Third, there were more older people in the NHL-first group at the diagnosis of the first tumor, which may relate to more comorbidities and lower tolerance to chemotherapy. Age is not only an important risk factor for the development of a second tumor, but also an established independent prognostic factor for MPMNs [4, 26, 27].

Our study presents the concomitant relationship between NHL and solid tumors to some extent with the help of a larger sample size and highlights DPMN as a clinical issue of concern. However, there are also limitations. It is a single-center retrospective study, future studies should include genetic sequencing to elucidate the pathogenesis and help guide treatment decisions.

## Conclusion

Patients with DPMNs comprising NHL and malignant solid tumors were not uncommon. In the NHL-first group, the interval time between the two tumors was shorter, and more lymphomas originating from extranodal sites were observed. Furthermore, patients with DPMNs diagnosed with NHL first exhibited a poorer prognosis, necessitating close monitoring and follow-up. Radiotherapy and chemotherapy were not always important risk factors for DPMNs. Extranodal NHL was more likely to develop solid tumors than nodal NHL. Further genetic testing and bidirectional analysis are warranted to elucidate the association between the two types of tumors.

## Data Availability

The datasets generated during and/or analysed during the current study are available from the corresponding author on reasonable request.
